# In Memoriam—Samuel Lilienthal, M. D.

**Published:** 1891-12

**Authors:** 


### IN MEMORIAM.

### SAMUEL LILIENTHAL, M. D.

At the first monthly meeting of the Alumnæ Association of the New York Medical College and Hospital for Women, held Oct. 22d, 1891, the following resolutions were adopted:

WHEREAS, It has pleased God to remove from us Dr. Samuel Lilienthal, who for twenty years was Professor in the New York Medical College and Hospital for Women;

"*Resolved*, That by the death of Dr. Lilienthal we have lost a wise counselor, a reliable friend, a sincere advocate of women in the medical profession, and one whose whole life was full of charity and good works among the sick and suffering;

"*Resolved*, That as a Professor, he was broad-minded while conservative, thorough, and scientific;

"*Resolved*, That as a physician, he always practiced and lived up to the highest standard of Homœopathy, calling himself "an humble disciple of the great Hahnemann;"

"*Resolved*, That as a scientific man, our school has lost an untiring student and teacher, and as a writer, our medical literature has lost its best translator and leader;

"*Resolved*, That these resolutions be entered upon the minutes of the Association and a copy sent to the family and to the medical journals.

Committee.

JULIA E. BRADNOR, M. D.

HARRIET C. KEATINGE, M. D.

ELIZABETH CLARKE, M. D.

M. BELLE BROWN, M. D.

